# Supplementary material for: Unlocking hidden biomolecular conformational landscapes in diffusion models at inference time
Source: ArXiv. 2026 Feb 4:arXiv:2512.03312v2. Originally published 2025 Dec 2. Preprint. [Version 2] (PMC12687860)
Supplement: Supplement 1 [file NIHPP2512.03312v2-supplement-1.pdf]

Supplementary Information

In the pages below we provide additional figures and code for key sections of our paper. We further refer the reader interested in code to the Boltz (`https://github.com/jwohlwend/boltz/tree/main`) and BioEmu (`https://github.com/microsoft/bioemu/tree/main`) repositories, as well as the reference twisted diffusion implementation at `https://github.com/blt2114/twisted_diffusion_sampler` and to the reference MBAR implementation at `https://pymbar.readthedocs.io/en/master/index.html`.

A Datasets

- **Domain motion:** 38 proteins consisting of the non-overlapping set of 22 domain motion proteins curated by [17] and 23 open-closed (OC23) conformation proteins curated by [13].
- **Membrane transporters:** 15 proteins from the transporter protein set (TP16) curated by [13]. One protein, SPF1, was excluded for compute considerations due to its long sequenve length.
- **Cryptic pockets:** 33 proteins that undergo cryptic pocket formation curated by [17]. RMSD metrics were computed using the cryptic pocket region defined by [17] . One protein, Q16539, was excluded because the region definitions were not available.
- **Fold switching:** 15 proteins that exhibit fold switching were randomly selected from the set of proteins curated by [27] and used to benchmark other conformational prediction methods including CF-random ([15]).

B Computational resources and timing

Compute for this work was run primarily on a cluster of 8 A40 GPUs. We typically generate 800 samples per protein for ConforMixRMSD, although motions of interest are typically visible more quickly. A rapid scan with a small number of samples per RMSD finishes in minutes.

We benchmarked wall clock sampling speeds for three proteins on a single A40 GPU without twisting (i.e., default sampling) and with twisting (i.e., ConforMix), for different numbers of simultaneous samples/particles. Because ConforMix modifies only the diffusion module and not the encoder, we separately time these modules. Total Time = encoder + decoder together. The penalty for running twisted diffusion at every timestep of the decoder is 2-3x wallclock speed. We believe our results demonstrate that the generated samples are substantially more informative than those from default sampling.

There is flexibility to tune performance by scheduling guidance and/or resampling only at certain timesteps of the decoder. We have not thoroughly explored the tradeoffs involved.

C MBAR

In this section we outline the Multistate Bennett Acceptance Ratio algorithm for free energy estimation introduced in Section 3.3. The input to MBAR is samples generated from ConforMix, i.e. collected from a series of conditional probability distributions pj (x|s), which are distinct from the unconditional distribution p(x|s). For notational convenience, we drop s in the remainder of this section, and we will write $p_j(x) = p(x|y_j)$, where y_j is the condition we impose.

Suppose we have a set of distributions $\{p(x|y_1), \cdots, p(x|y_J)\}$ and from each $p(x|y_j)$ we draw i.i.d. a set of n_j samples $\{x_i^{(j)}\}$. The key principle of MBAR is to take advantage of overlap between the distributions. Although a given sample $x_i^{(j)}$ was generated from its distribution $p(x|y_j)$, it is equally correct to model the entire set of samples $\{x_i^{(j)}\}$ as generated from the mixture distribution $p_{mix}(x) = \sum_{j\in\{1,\cdots,J\}} q(j)p(x|y_j)$, where we define $q(j) = \frac{n_j}{\sum_l n_l}$ to be the fraction of samples collected at j. To estimate the partition functions, we now write the importance sampling estimate for an arbitrary observable $O(x)$ in an arbitrary ensemble $p(x|y_j)$, given samples from the mixture $p_{mix}(x)$:

Table 2: Runtime analysis of ConforMix with Boltz

Protein	# Residues	# Atoms	# Simult. Samples	Diffusion Time (s)		Total Time (s)
				default	ConforMix	default
P0DP23	148	1184	1	6.9 ± 0.2	-	9.5 ± 0.1
			2	-	28.1 ± 1.3	-
			4	9.7 ± 0.0	31.6 ± 0.7	12.3 ± 0.0
			16	31.7 ± 0.1	71.0 ± 0.4	34.3 ± 0.1
P37487	309	2400	1	7.2 ± 0.2	-	18.7 ± 0.2
			2	-	30.7 ± 0.7	-
			4	20.0 ± 0.2	44.4 ± 0.2	31.5 ± 0.2
			16	70.7 ± 0.1	143.9 ± 0.6	82.1 ± 0.1
B0F0C5	497	4000	1	11.5 ± 0.0	-	47.2 ± 0.0
			2	-	48.6 ± 0.1	-
			4	37.4 ± 0.2	86.9 ± 0.7	73.1 ± 0.2
			16	134.6 ± 0.1	258.9 ± 0.3	170.4 ± 0.1

Runtime speed of Boltz and ConforMix-Boltz for three protein systems. Columns: diffusion only and encoder + diffusion for three protein systems, in seconds. # Simultaneous Samples is the number of samples moving through diffusion, either independently of each other (default sampling) or with guidance and resampling (ConforMix). Standard deviation is shown over 5 runs.

$$\int O(x)p(x|y_j)dx = \int O(x)\frac{p_{mix}(x)}{p_{mix}(x)}p(x|y_j)dx \approx \left\langle O(x)\frac{p(x|y_j)}{p_{mix}(x)}\right\rangle_{mix} \quad (3)$$

Let us choose $O(x) = 1$ for all x. Then we have

$$1 = \left\langle \frac{p(x|y_j)}{p_{mix}(x)}\right\rangle_{mix} = \left\langle \frac{p(x|y_j)}{\sum_{r\in\{1,\cdots,J\}} q(r)p(x|y_r)}\right\rangle_{mix}. \quad (4)$$

Turning to the conditional distributions $p_j(x)$ sampled by ConforMix, we previously defined the twisting potential $y_j(x) = \exp(-k_j(g_j(x) - g_j^{(0)}))/Z_j$, where $g_j(x)$ is an observable. We then have

$$p(x|y_j) = \frac{p(x)y_j(x)}{p(y_j)} = \frac{p(x)\exp\left(-k_j\left(g_j(x) - g_j^{(0)}\right)\right)/Z_j}{p(y_j)}. \quad (5)$$

Substituting this expression into the numerator and denominator of (4) and rearranging, we obtain

$$p(y_j) \approx \left\langle \frac{\exp\left(-k_j\left(g_j(x) - g_j^{(0)}\right)\right)/Z_j}{\sum_{r\in\{1,\cdots,J\}} p(r)\left(\frac{\exp\left(-k_r\left(g_r(x)-g_r^{(0)}\right)\right)}{Z_r p(y_r)}\right)}\right\rangle_{mix}. \quad (6)$$

Crucially, $p(x)$ cancels, meaning we do not need to evaluate the absolute probability of a given sample under the diffusion model. Finally, observe that the unconditional distribution $p(x)$, the target of our reweighting, can also be written as $p(x|y_0)$ where $k_0, g_0, g_0^{(0)} = 0$. Thus, for samples collected from J biased distributions, we form a system of $J+1$ equations in $J+1$ unknowns by writing Equation 6 for each $p(x|y_j)$ plus $p(x|y_0) = p(x)$. This system can be solved numerically to obtain the target partition functions. In our use case, J is typically on the order of 10 to 100, although MBAR can accommodate larger sets if necessary.

D Benchmarking methods for ConforMixRMSD

In Section 4.1 we compare ConforMixRMSD performance to default Boltz sampling and to three other methods: AFCluster, AFsample2, and CF-random. To allow for an apples-to-apples comparison, we reimplement the key steps of each of these methods to produce inputs for Boltz.

For AFsample2, we use the recommended settings of 1000 randomly generated input MSAs with the column randomization fraction set to 0.15. The published CF-random protocol modifies the `-max-seq` and `-max-extra-seq` inputs to AlphaFold 3, but Boltz lacks the `-max-extra-seq` input, so the MSA modifications we make are equivalent to only the first option. We use the suggested settings of `-max-seq = 1,2,4,8,16,32,64,128,256,512` with 50 randomly sampled MSAs in each condition, for a total of 500 CF-random input MSAs per protein system. We run AFCluster with default settings; the number of clusters, and therefore the number of sampled MSAs per system, varies per system depending on the initial MSA.

E Outliers

To assess the structural validity of the generated samples, we examined outliers within the generated samples from the domain motion protein set using the Boltz implementation.

Table 3: Outlier fractions for structural quality metrics by method

	**Bond Lengths**	**Bond Angles**	**Ramachandran Angles**
Default Boltz sampling	2.96×10^{-3}	2.14×10^{-4}	6.63×10^{-4}
AFCluster-Boltz	5.60×10^{-3}	6.02×10^{-4}	1.51×10^{-3}
AFsample2-Boltz	1.08×10^{-2}	2.94×10^{-4}	2.13×10^{-3}
CF-random-Boltz	1.08×10^{-2}	2.94×10^{-4}	2.13×10^{-3}
ConforMixRMSD-Boltz (unfiltered)	9.52×10^{-2}	1.68×10^{-2}	1.79×10^{-3}
ConforMixRMSD-Boltz (filtered)	9.12×10^{-2}	1.42×10^{-2}	1.09×10^{-3}

Each entry in the table is the total fraction of bonds/angles/dihedrals marked as outliers, across all sampled frames and proteins.

Outlier analysis was performed using default settings of the `ramalyze` module of the `cctbx` toolkit, associated with Phenix ([18]), to identify Ramachandran outliers. We also evaluate backbone bond length and bond angle outliers as deviations more than 4σ from reference values. We assess every generated frame for outliers and compute the total fraction of bonds/angles/dihedrals that are marked as outliers, across all frames and proteins (Table 3). While the fraction of outliers is somewhat elevated in ConforMix sampling, the vast majority of bond lengths, bonds angles, and Ramachandran angles are within physical parameters. ConforMix-generated samples are generally usable as is for visual examination or motion statistics. If needed, most outliers are easily resolvable with a brief relaxation, as is standard in the AlphaFold workflow. For ConforMix, we also report results after our filtering, since we intentionally push some proteins beyond their normal range of flexibility in order to capture the full range of motion and reject unphysical samples afterwards.

F Twisted diffusion sampling algorithm

The subroutine of twisted diffusion sampling is described below. The WEIGHT function accounts for the effects of the bias potential, and RESAMPLE outputs a new subset of the particles based on the weights. We use systematic resampling in our implementation. A more complete description of the algorithm is provided in [38].

G Free Energy Estimation with BioEmu details

The `trp-cage-helix` free energy estimation task described in the main text was performed using our implementation of ConforMix-BioEmu. The sampling procedure is similar to ConforMixRMSD (Algorithm 1), except that (1) we use the experimentally determined structure as a reference, and (2) we omit the secondary structure masking. This setup mimics common tasks in quantitative estimation of free energies, where one or more reference states can be described. For the particular free energy task described (estimation of ΔG between 3Å and 7.5Å RMSD to the reference structure), we construct guidance potentials with an RMSD spacing of 0.5Å, between 2.0Å and 8.5Å from the

Algorithm 2 Twisted diffusion sampler for conformational generation

Input: Guidance potential $y(x)$, input system s, noise schedule $\{\sigma_t\}$, guidance scale schedule $\{\epsilon_t\}$, number of particles to generate $N_{particles}$

Output: Samples satisfying $\textsc{Model}(x|y, s)$

for $i = 1, \cdots, N_{particles}$ **do**
  $x_i^T \in \mathbb{R}^{n_{atoms} \times 3} \sim \mathcal{N}(\vec{0}, \sigma_T \cdot I_3)$
  $w_i \leftarrow \textsc{Weight}(x_i^T)$
end for
for $t = T - 1, \cdots, 0$ **do**
  for $i = 1, \cdots, N_{particles}$ **do**
    $\hat{x}_i^{t+1} \leftarrow \textsc{StructureDiffusionModule}(x_i^{t+1}, t + 1)$ *// prediction of* x_0 *at step* $t + 1$
    $bias_i^{t+1} = y(\hat{x}_i^{t+1})$
    $x_i^t \leftarrow x_i^{t+1} + \sigma_t(\hat{x}_i^{t+1} - x_i^{t+1}) + \epsilon_t(\nabla_{x_i^{t+1}} \log bias_i^{t+1})$ *// backprop through model*
    $w_i \leftarrow \textsc{Weight}(x^t)$
  end for
  $x_{1:N}^t \sim \textsc{Resample}(x_{1:N}^t; w_{1:N})$
end for
return $\{x_i^0\}_{i=1}^{N_{particles}}$

reference. We collect 60 samples at each potential and perform free energy estimation with MBAR, as described in the text.

A tunable parameter in twisted diffusion sampling is the accuracy of the approximation $\hat{x}_i^{t+1}$. Because free energy estimation demands more accurate sampling than many qualitative tasks, we find it advantageous to substitute a multi-step denoised approximation (approximately 5 denoising steps) for the typical first- or second-order approximation used in the rest of our work. The performance cost of this approximation is on the order of a factor of 2. Because ConforMix free energy estimation aids in evaluating rare states, this penalty is still small compared to the alternative of running default sampling.

H Supplemental Data and Figures

Table 4: AUC scores for coverage of experimentally-determined reference conformations by method and dataset

		Domain motion (n=38)	Membrane transporters (n=15)	Cryptic pockets (n=31)	Fold switching (n=15)
**Worst-matched reference conformation** (harder task)	Default Boltz sampling	0.37 (±0.09)	0.16 (±0.11)	0.23 (±0.08)	0.13 (±0.10)
	AFCluster-Boltz	0.45 (±0.09)	0.26 (±0.12)	0.42 (±0.09)	0.22 (±0.13)
	CF-random-Boltz	0.52 (±0.09)	**0.28 (±0.13)**	**0.43 (±0.10)**	**0.24 (±0.12)**
	AFsample2-Boltz	0.47 (±0.09)	0.26 (±0.14)	0.35 (±0.10)	0.16 (±0.13)
	**ConforMixRMSD-Boltz**	**0.61 (±0.08)**	**0.28 (±0.13)**	**0.43 (±0.09)**	0.22 (±0.11)
**Best-matched reference conformation** (easier task)	Default Boltz Sampling	**0.77 (±0.04)**	0.49 (±0.14)	0.80 (±0.05)	0.54 (±0.19)
	AFCluster-Boltz	0.73 (±0.06)	0.49 (±0.14)	**0.82 (±0.03)**	0.50 (±0.16)
	CF-random-Boltz	0.73 (±0.07)	0.54 (±0.11)	0.81 (±0.06)	0.54 (±0.17)
	AFsample2-Boltz	0.73 (±0.06)	0.50 (±0.14)	0.79 (±0.06)	**0.57 (±0.20)**
	**ConforMixRMSD-Boltz**	**0.77 (±0.04)**	**0.56 (±0.12)**	0.80 (±0.04)	**0.57 (±0.16)**

Coverage measures the fraction of proteins with samples matching a reference conformation within X% of the RMSD between reference structures. Displayed is the **area under the coverage curve, AUC** integrated from 0-100% of the reference-to-reference RMSD. Error bars are 95% confidence intervals over 1,000 bootstraps.

We evaluate coverage separately for best-matched and alternate (worst-matched) states. We use X% of the RMSD between reference structures, rather than RMSD directly, to normalize for the magnitude of each protein's conformational transition.

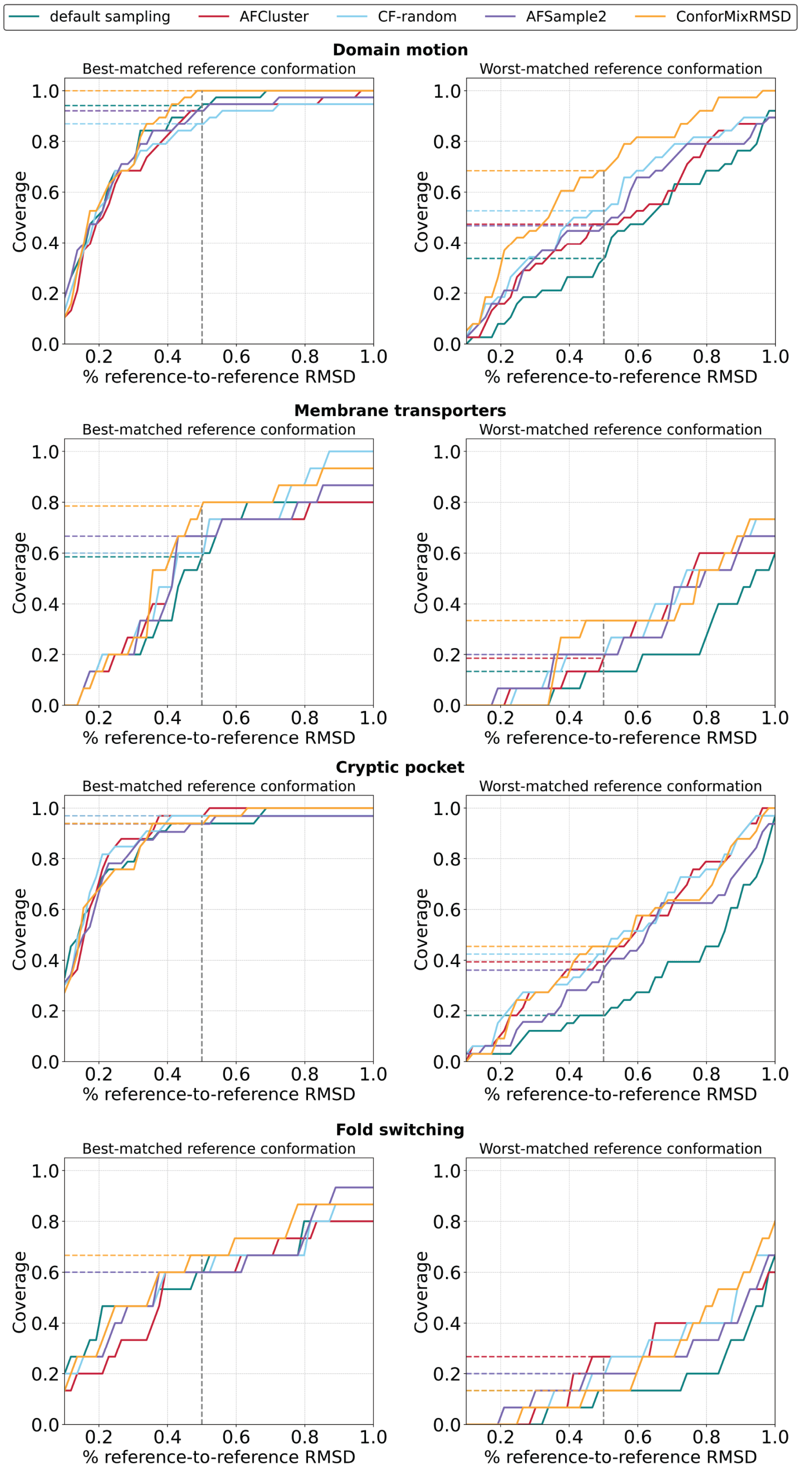

Figure S1: Coverage of experimentally observed states across methods and datasets. Coverage measure the fraction of proteins with samples matching a reference conformation within X% of the RMSD between reference structures. For each protein, we compute the RMSD of the closest sampled structure to each reference conformation. We then separate these into two groups: the best-matched reference (lower RMSD match) and the alternate reference (higher RMSD match). We aggregate performance across each dataset, evaluating coverage separately for best-matched and alternate (worst-matched) states. Coverage is computed at thresholds of X% of the RMSD between reference structures, normalizing for the magnitude of each protein's conformational transition.

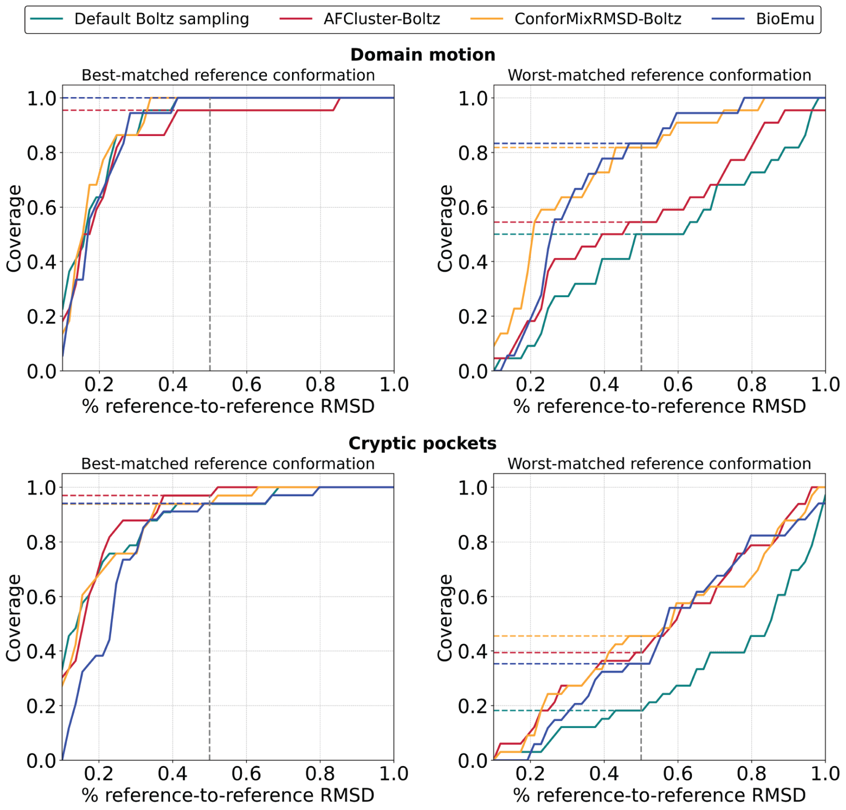

Figure S2: Comparison of coverage of experimentally observed states to BioEmu. Coverage measure the fraction of proteins with samples matching a reference conformation within X% of the RMSD between reference structures. For each protein, we compute the RMSD of the closest sampled structure to each reference conformation. We then separate these into two groups: the best-matched reference (lower RMSD match) and the alternate reference (higher RMSD match). We aggregate performance across each dataset, evaluating coverage separately for best-matched and alternate (worst-matched) states. Coverage is computed at thresholds of X% of the RMSD between reference structures, normalizing for the magnitude of each protein's conformational transition.

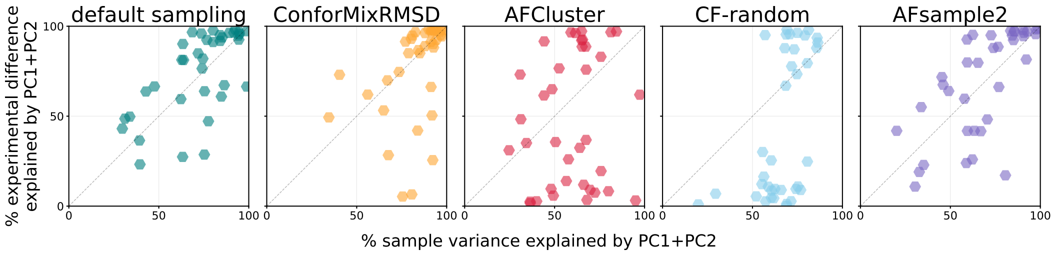

Figure S3: Principal component analysis of domain motion protein set, with extended benchmarks. Principal component analysis was performed pairwise $C\alpha$ distances of structures sampled from each method. Each point describes results for one protein.

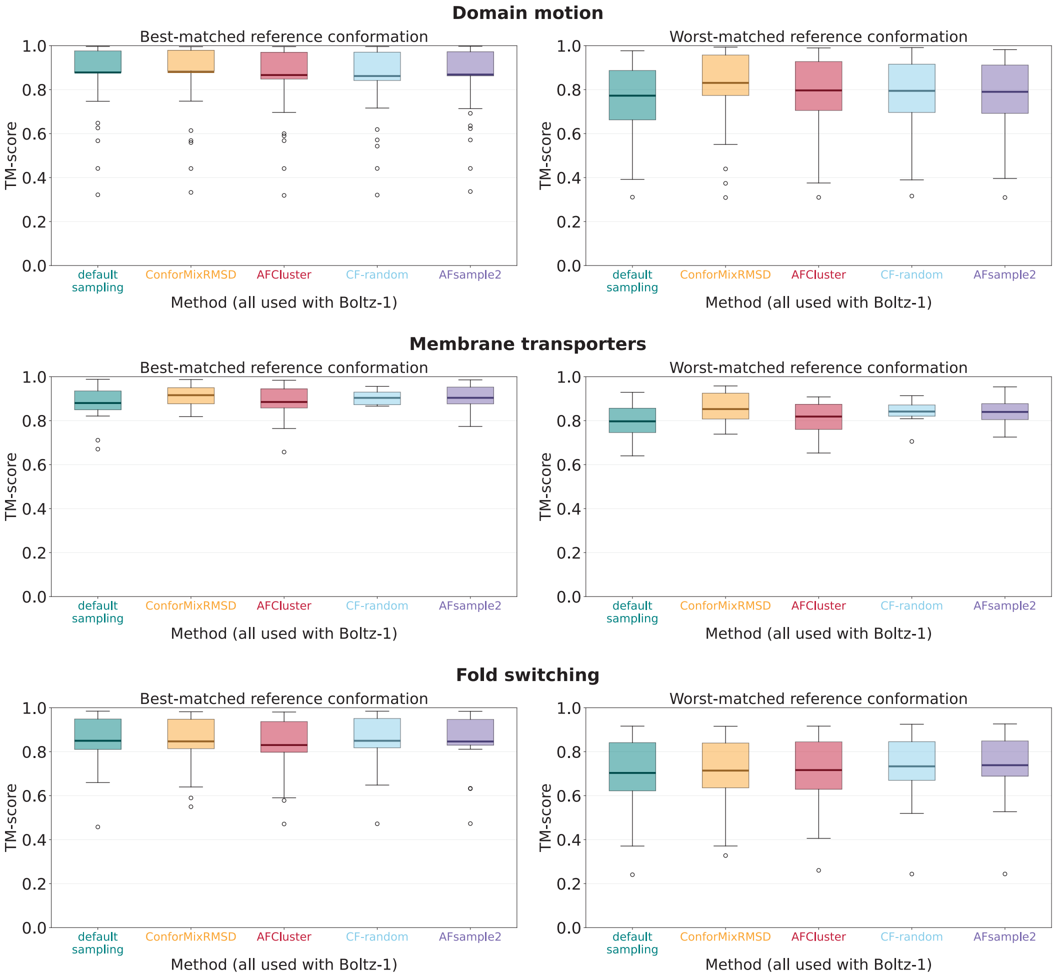

Figure S4: TM-score analysis across methods and datasets. TM-score is a length-independent structural similarity metric (0 − 1 scale), with higher values indicating closer structural matches.

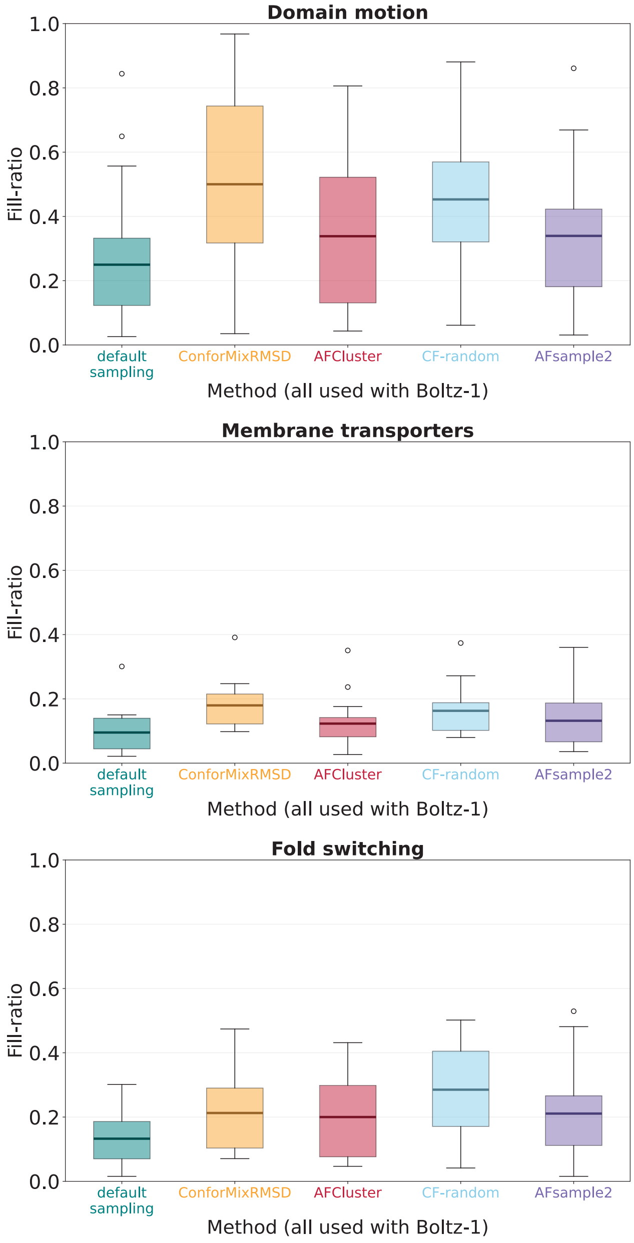

Figure S5: Fill ratio analysis across methods and datasets. The fill ratio, developed in [13], is a TM-score–based metric that quantifies the amount of structure diversity *between* reference states that is captured.

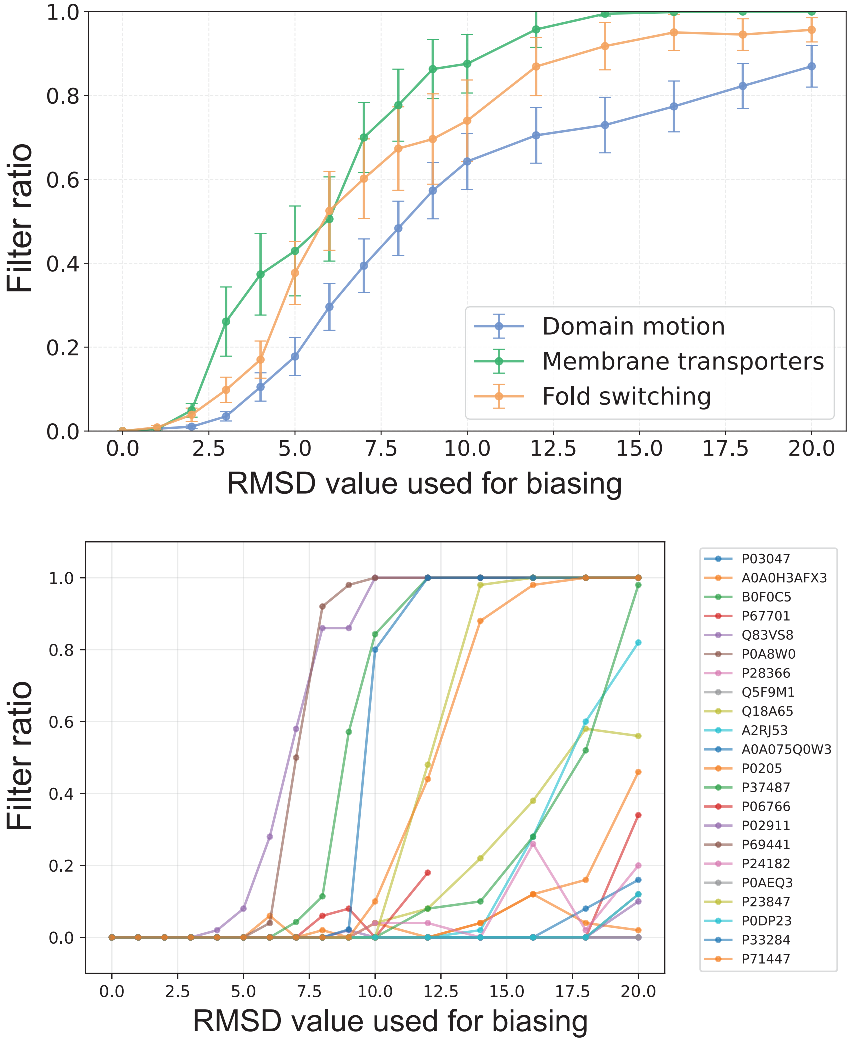

Figure S6: Filtering based on plDDT scores with a 10-residue sliding rejects more samples as structure generation is biased further away from the default sample. The percent of ConforMixRMSD samples that are filtered out is plotted against the RMSD value against the default sample that used to condition sampling. Top: mean filter ratio across all proteins in each dataset. Error bars show the standard error of the mean. Bottom: filter ratio curves for a subset of domain motion proteins. Proteins tolerate varying amounts of biasing away from the default sample.

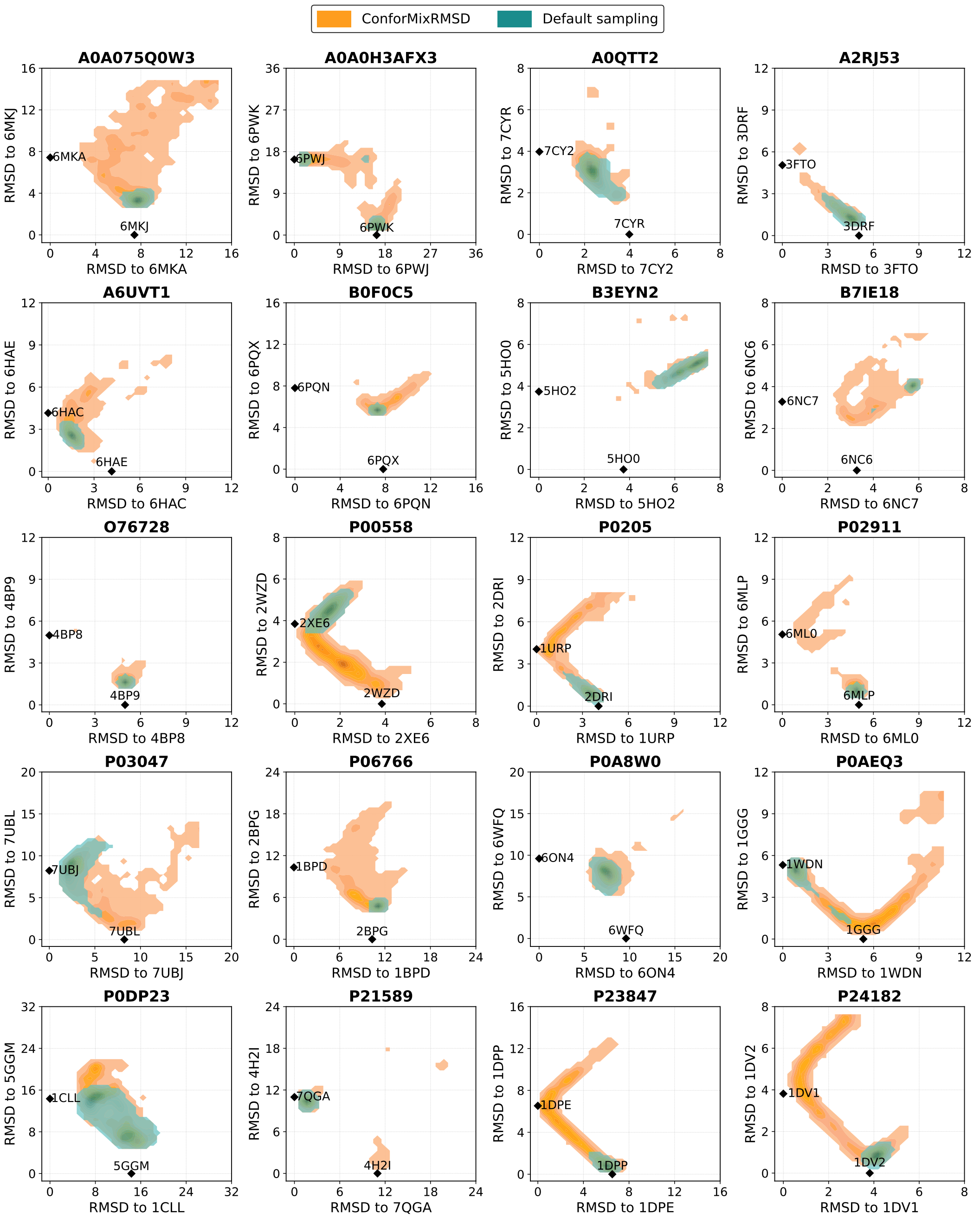

Figure S7: Density of sampling relative to reference experimentally-determined structures for **domain motion** dataset.

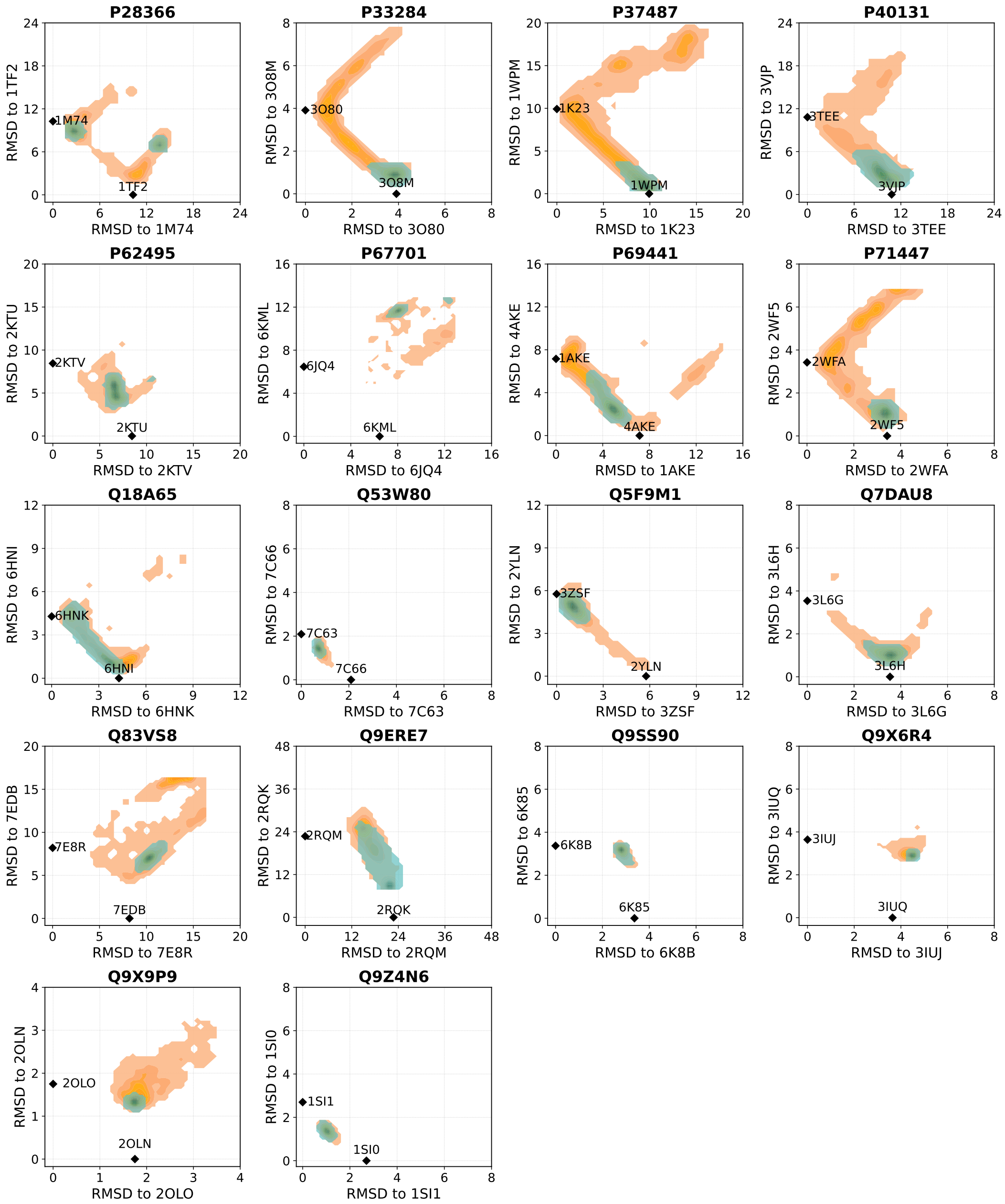

Figure S7: Density of sampling relative to reference experimentally-determined structures for **domain motion** dataset.

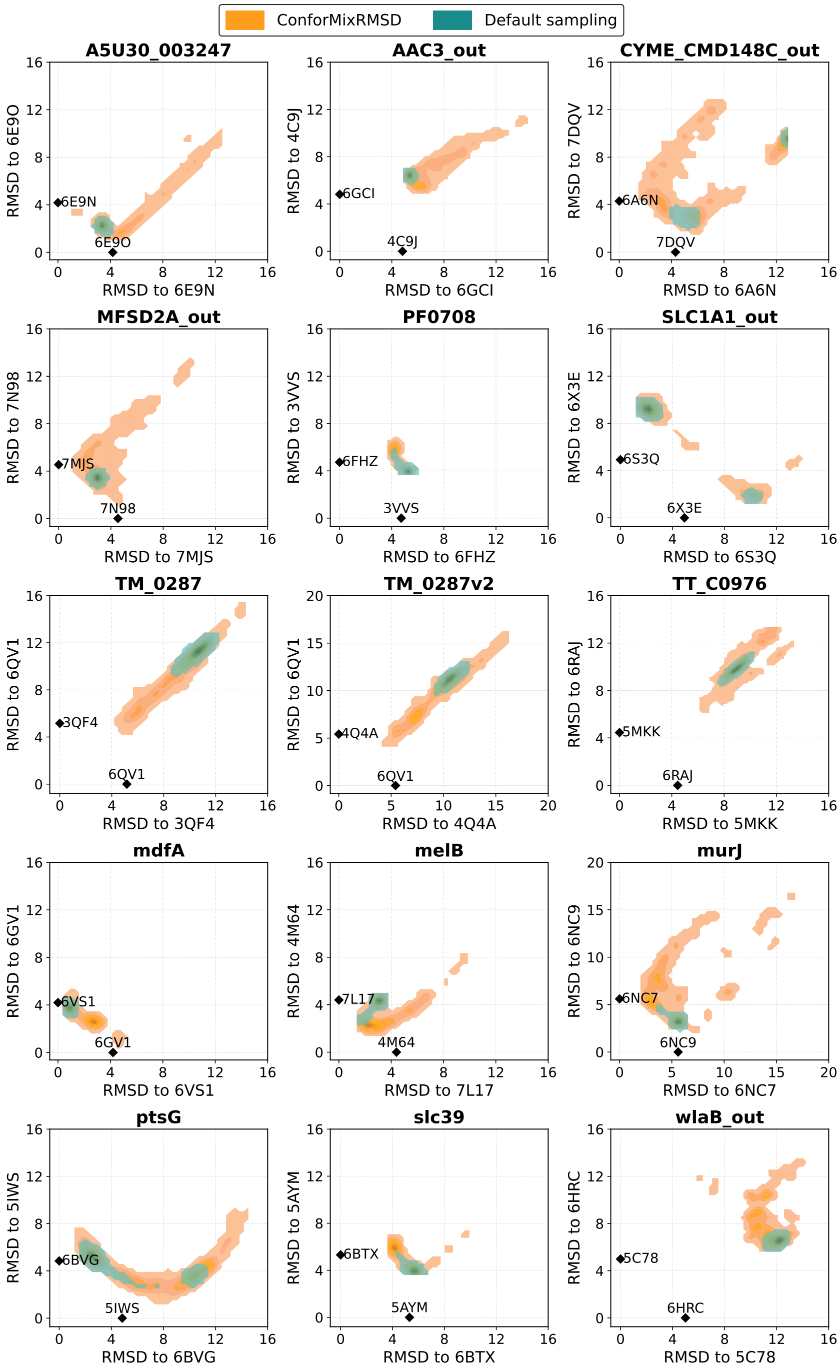

Figure S8: Density of sampling relative to reference experimentally-determined structures for **membrane transporters** dataset.

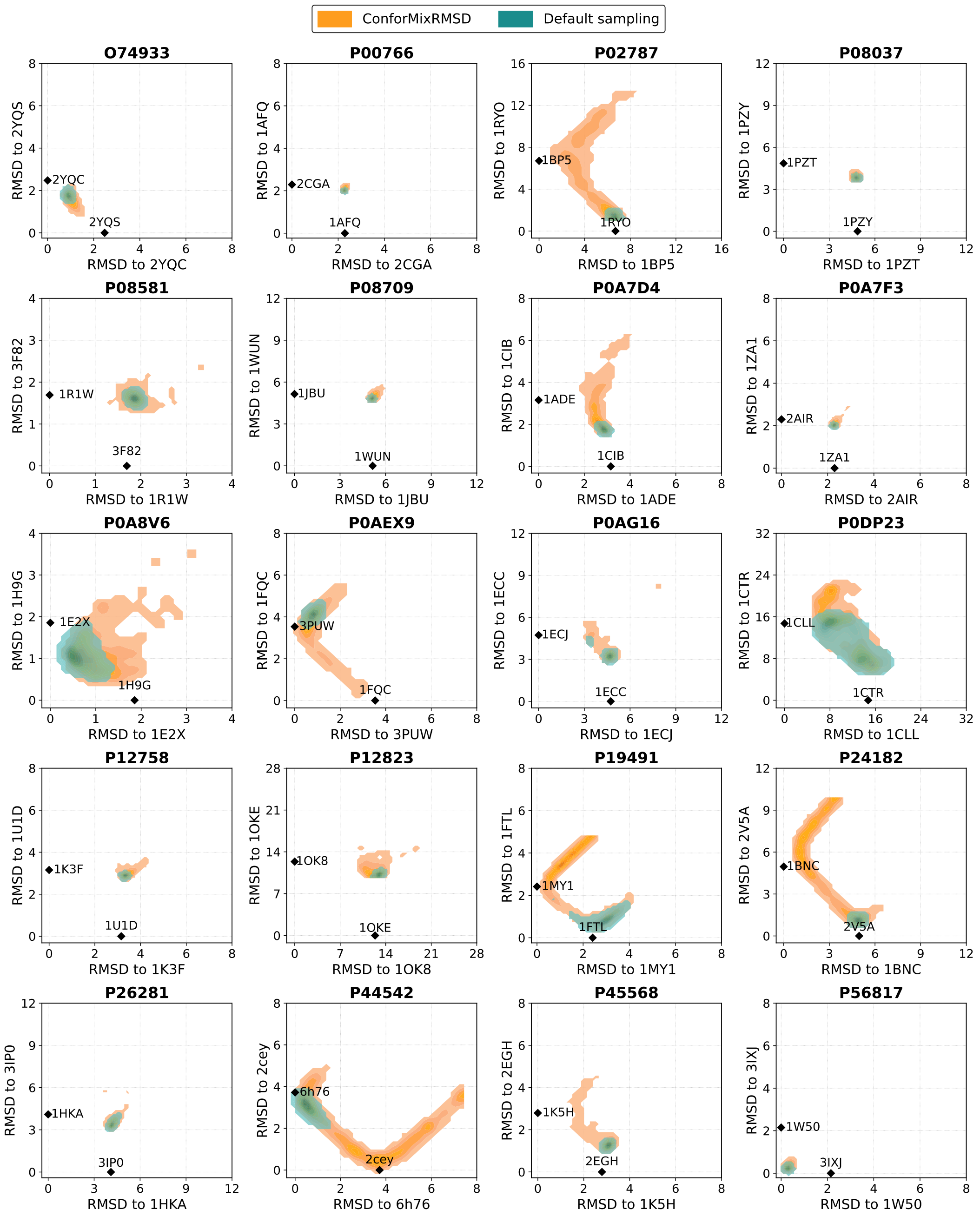

Figure S9: Density of sampling relative to reference experimentally-determined structures for **cryptic pocket** dataset.

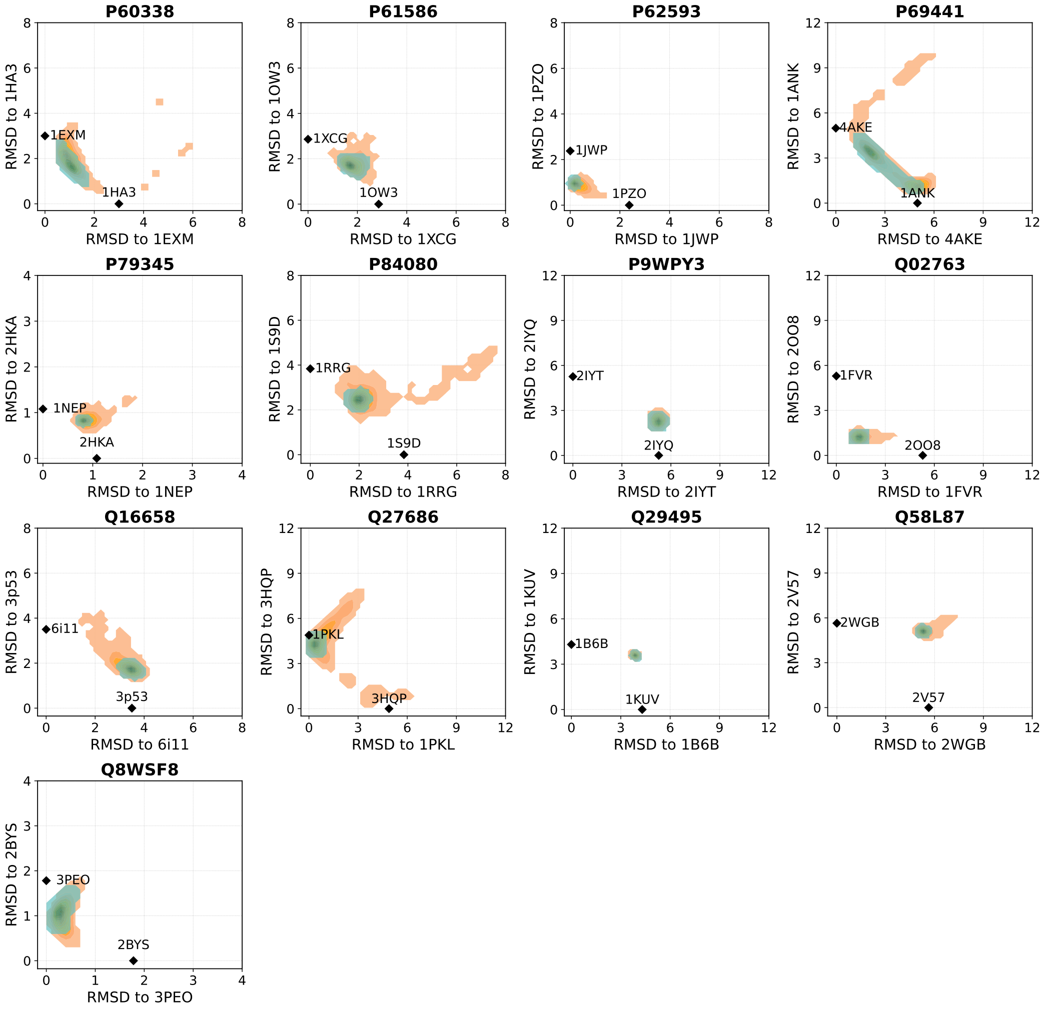

Figure S9: Density of sampling relative to reference experimentally-determined structures for **cryptic pocket** dataset.

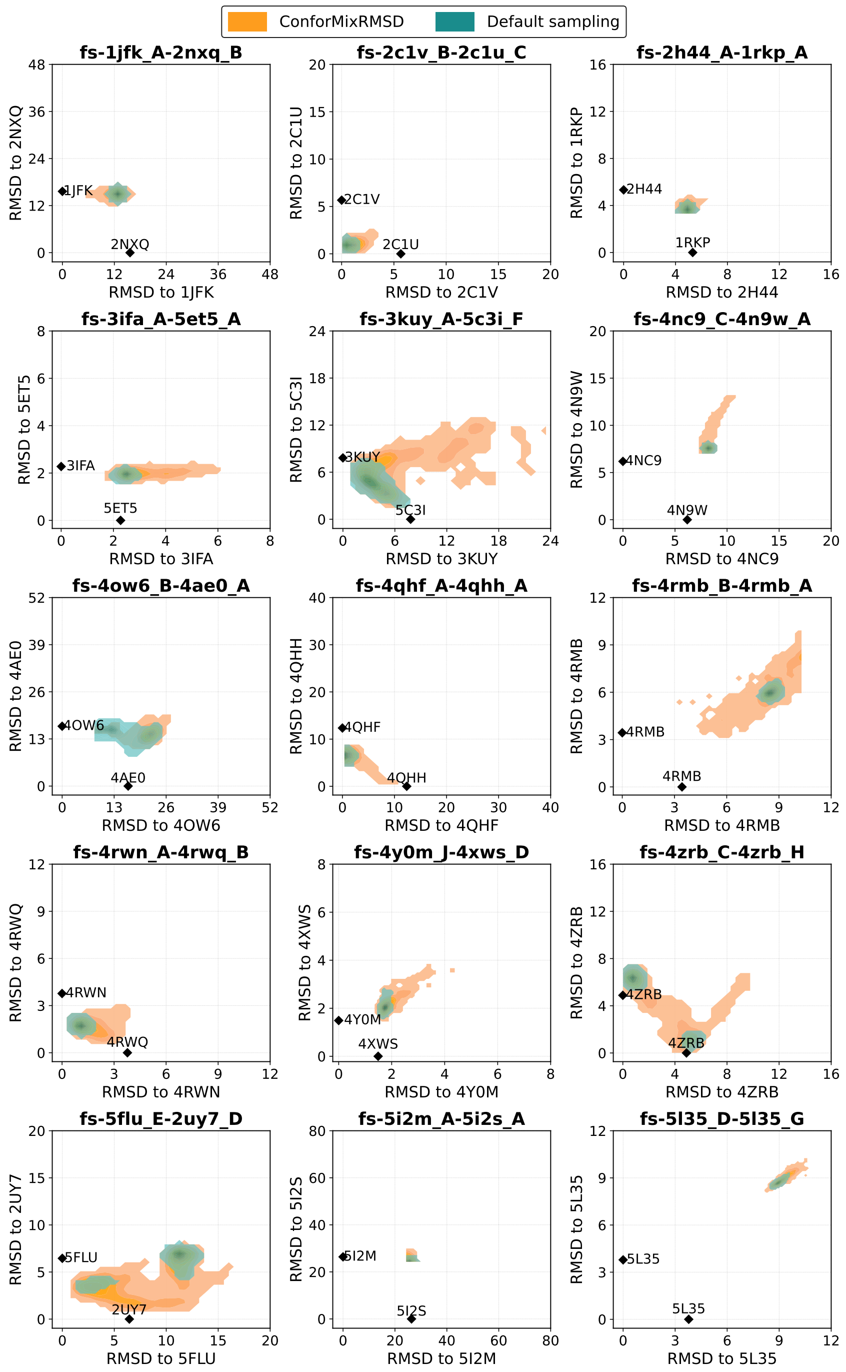

Figure S10: Density of sampling relative to reference experimentally-determined structures for **fold switching** dataset.

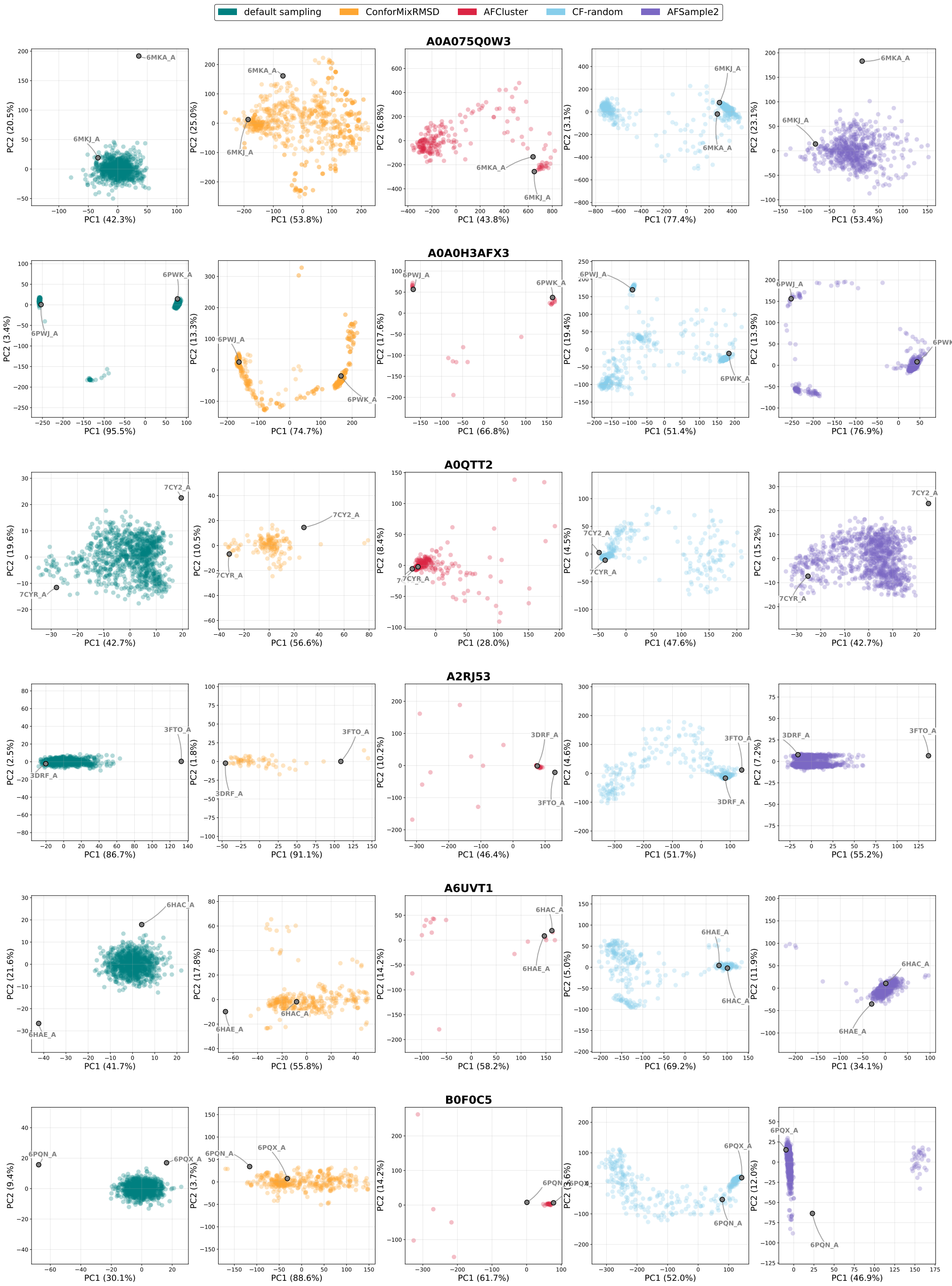

Figure S11: Principal component analysis of domain motion proteins.

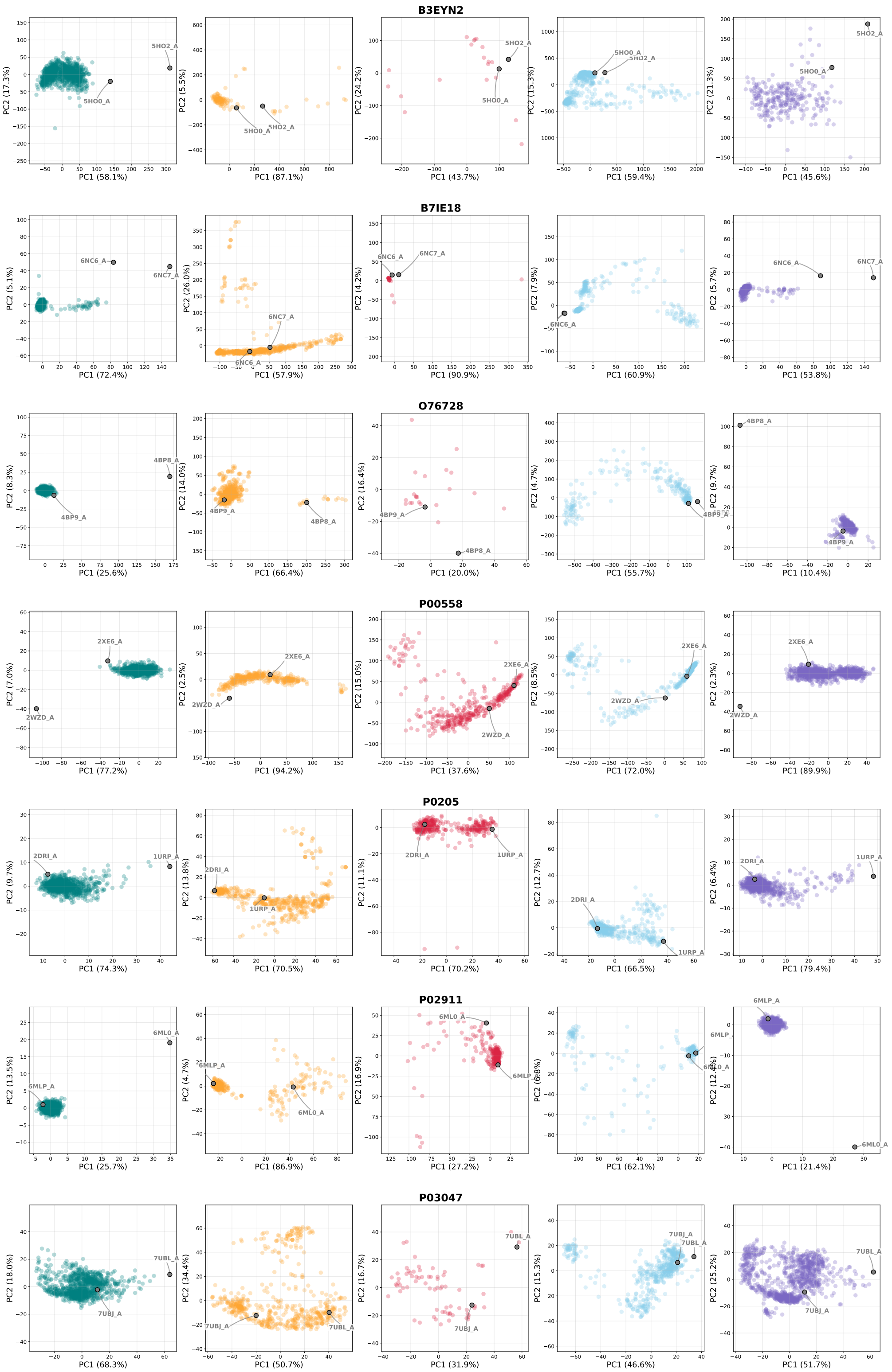

Figure S11: Principal component analysis of domain motion proteins.

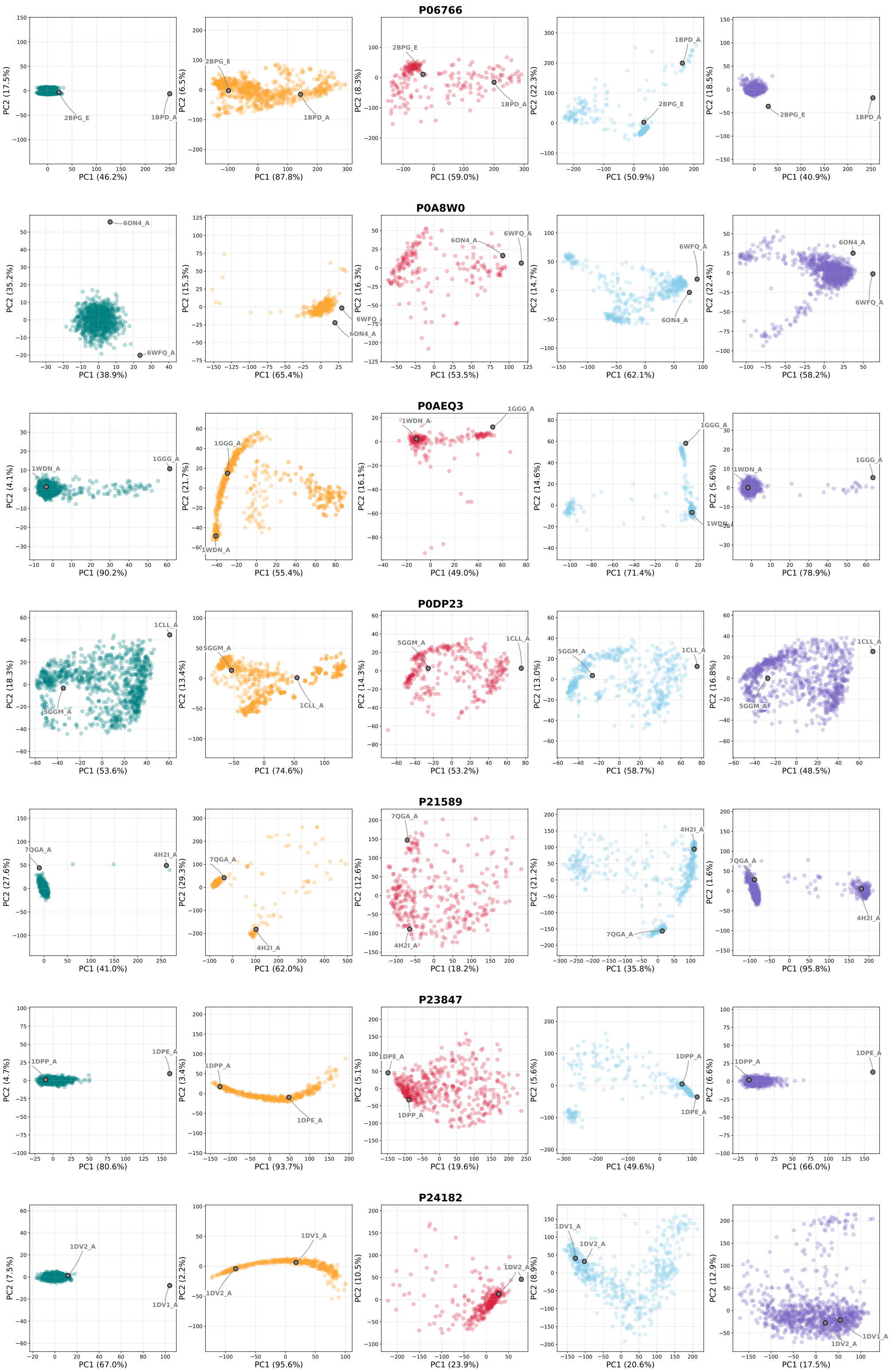

Figure S11: Principal component analysis of domain motion proteins.

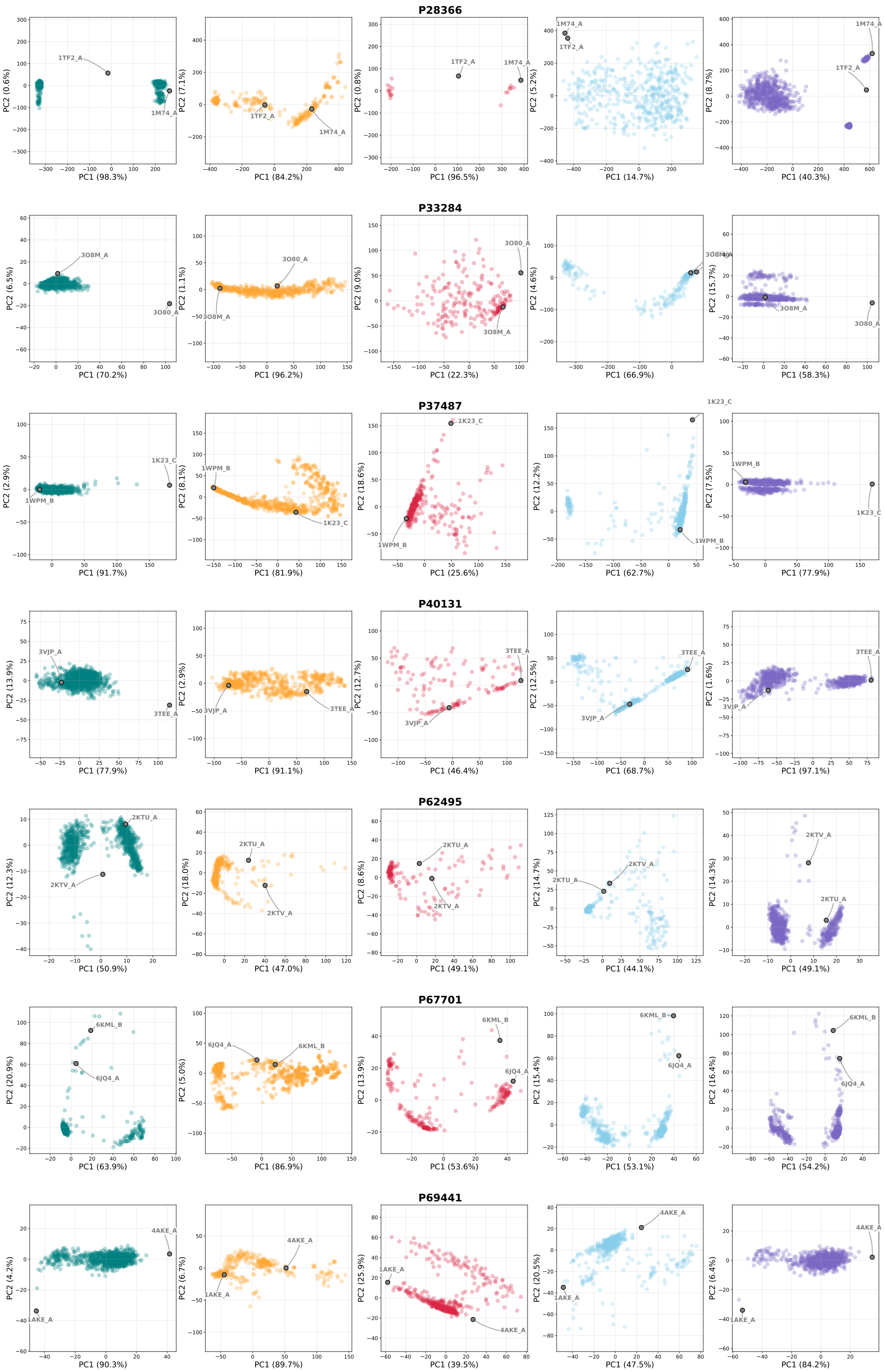

Figure S11: Principal component analysis of domain motion proteins.

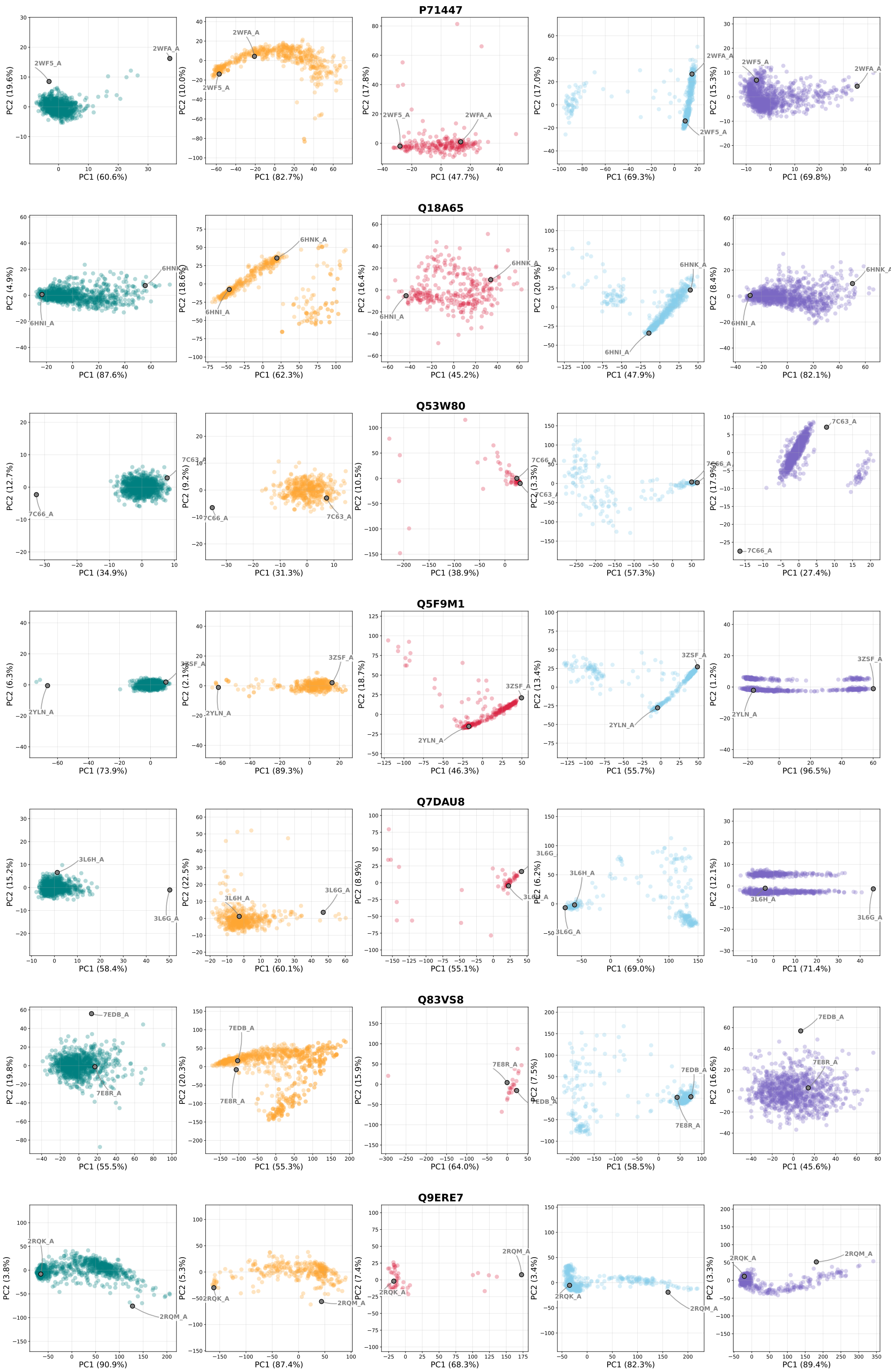

Figure S11: Principal component analysis of domain motion proteins.

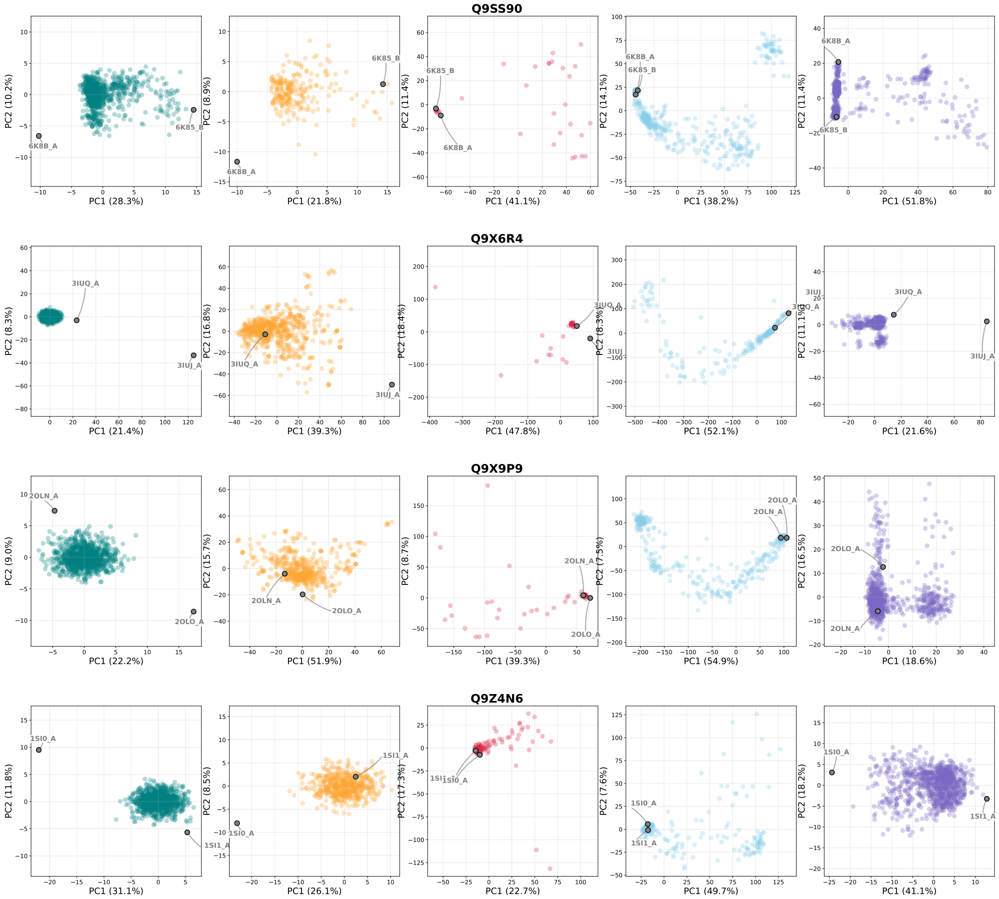

Figure S11: Principal component analysis of domain motion proteins.
